# The Lateralization of Intrinsic Networks in the Aging Brain Implicates the Effects of Cognitive Training

**DOI:** 10.3389/fnagi.2016.00032

**Published:** 2016-03-03

**Authors:** Cheng Luo, Xingxing Zhang, Xinyi Cao, Yulong Gan, Ting Li, Yan Cheng, Weifang Cao, Lijuan Jiang, Dezhong Yao, Chunbo Li

**Affiliations:** ^1^Key Laboratory for NeuroInformation of Ministry of Education, High-Field Magnetic Resonance Brain Imaging Key Laboratory of Sichuan Province, Center for Information in Medicine, School of Life Science and Technology, University of Electronic Science and Technology of ChinaChengdu, China; ^2^Shanghai Key Laboratory of Psychotic Disorders, Shanghai Mental Health Center, Shanghai Jiao Tong University School of MedicineShanghai, China; ^3^Changning Mental Health CenterShanghai, China; ^4^Bio-X Institutes, Key Laboratory for the Genetics of Developmental and Neuropsychiatric Disorders, Ministry of Education, Shanghai Jiao Tong UniversityShanghai, China

**Keywords:** aging, fMRI, lateralization, functional network, cognitive training

## Abstract

Lateralization of function is an important organization of the human brain. The distribution of intrinsic networks in the resting brain is strongly related to cognitive function, gender and age. In this study, a longitudinal design with 1 year’s duration was used to evaluate the cognitive training effects on the lateralization of intrinsic networks among healthy older adults. The subjects were divided into two groups randomly: one with multi-domain cognitive training over 3 months and the other as a wait-list control group. Resting state fMRI data were acquired before training and 1 year after training. We analyzed the functional lateralization in 10 common resting state fMRI networks. We observed statically significant training effects on the lateralization of two important RSNs related to high-level cognition: right- and left- frontoparietal networks (FPNs). The lateralization of the left-FPN was retained especially well in the training group but decreased in the control group. The increased lateralization with aging was observed in the cerebellum network (CereN), in which the lateralization was significantly increased in the control group, although the same change tendency was observed in the training group. These findings indicate that the lateralization of the high-level cognitive intrinsic networks is sensitive to multi-domain cognitive training. This study provides neuroimaging evidence to support the hypothesis that cognitive training should have an advantage in preventing cognitive decline in healthy older adults.

## Introduction

The human brain is the most complex system in nature. The interhemispheric interaction is crucial to brain functions such as motor and cognitive processes. However, hemispheric specialization is also an obvious organizing principle for efficient information processing, such as leftward lateralization for speech and rightward lateralization for visuospatial attention (Corballis, 2014). Postmortem and neuroimaging studies have displayed the asymmetries of the human brain (Goldberg et al., 2013). Recently, resting state functional magnetic resonance imaging (fMRI) has been widely used in the studies of the human brain in which it was organized into several intrinsic resting state networks (RSNs). More and more studies have provided the evidence to support the importance of these RSNs on the brain function, such as default mode and attention. In general, the spatial pattern of these RSNs is relatively stable across subjects. These RSNs demonstrate either spatial hemispheric symmetry, such as the default mode network (DMN) and sensorimotor network (SMN), or hemispheric asymmetry, such as the frontoparietal network (FPN) and attention network (Fox et al., 2006). Hemispheric symmetry, which is represented by the spatial lateralization of RSNs, may reflect the slight changes of RSNs in different states of the brain. For example, the lateralization is influenced by development (Kelly et al., 2009), gender (Agcaoglu et al., 2015) and diseases (Luo et al., 2014b). The lateralization of RSNs is also observed to be significantly altered in healthy older adults compared with younger adults (Agcaoglu et al., 2015; Seidler et al., 2015).

Neurodegeneration is associated with increasing age (Seidler et al., 2010), i.e., declining cognition and decreases in motor performance (Cheng et al., 2012; Brown et al., 2013). Physical activity and exercise have positive influences on neurodegeneration (Brown et al., 2013). It has been observed that directed cognitive training interventions will increase mental activity in older adults, helping them to resist age-related cognitive decline and even potentially reducing the risk of dementia (Gates and Valenzuela, 2010). In a previous behavioral study, we observed that cognitive training can improve multi-cognition in community-living older adults, including memory, attention and neuropsychological status (Cheng et al., 2012). However, the underlying brain mechanism allowing the training to enhance function has been unclear till now. Noninvasive neuroimaging techniques, especially fMRI, are used to investigate the association between the intervention and brain function. For example, musical training would enhance the functional connectivity in perceptual and motor systems (Luo et al., 2012a) and salience network (Luo et al., 2014a). Additionally, the motor-related exercise increased the integration of motor performance and imaging systems (Gong et al., 2015, 2016; Li et al., 2015a). MRI studies of aging adults have shown enlarged local cortex thickness and enhanced functional integration related to the expertise in cognitive and sensorimotor interventions (Boyke et al., 2008; Lustig et al., 2009; Cao et al., 2014; Li et al., 2014). Therefore, we hypothesized that the lateralization of RSNs found in resting state fMRI scanning might provide an approach to reflect the influence of cognitive training on healthy older adults.

In this study, to test our hypothesis, a longitudinal design with 1 year’s duration was used to evaluate the cognitive training effects on the lateralization of intrinsic networks among healthy older adults. Healthy older adults with community-living were recruited and divided into two groups randomly: one received a multi-domain cognitive training over 3 months; the other was included in a wait-list control group. Resting state fMRI data were acquired before training and 1 year after training. The functional lateralization of 10 common resting state fMRI networks was evaluated between two groups using repeated measures analysis of variance (ANOVA).

## Materials and Methods

### Participants

Forty healthy older adults were recruited from three community centers around Tongji Hospital in Shanghai via a dispatched notice/broadcasting by the local institute of community service from March 2008 to April 2008. All participants were admitted to the study after a personal interview according to the inclusion criteria as follows: normal functional capacity; independent living in the community; age (range: 65–75 years); educational level (more than 1 year); no abnormality in hearing, vision, or communication status; a score of 19 or above (the lower normal cut-off point of the MMSE score is due to the lower educational level in China than in the US; Li et al., 2006) using the Chinese version of the Mini-Mental State Examination (MMSE); and no physical disease or psychotic disorder. The subjects with obvious cognitive decline, a diagnosis of AD, a brain tumor, or serious neurological and/or psychiatric disorders such as major depressive disorder and schizophrenia were excluded in this study. All of participants underwent cognitive measurements and fMRI scanning at baseline and at 1 year after training. They were divided randomly into two groups, including the multi-domain training group (*n* = 23) and the control group (*n* = 17). One participant out of 23 in the multi-domain training group and one in the single-domain training group were excluded because of left-handedness, which would interrupt the study of the lateralization of brain (Mackey et al., 2013). This study was reviewed and approved by the Ethical Committee of the East China Normal University. This study was performed according to the recommendation of the Ethical Committee of the East China Normal University with written informed consent from all subjects. All subjects gave written informed consent in accordance with the Declaration of Helsinki.

### Cognitive Interventions and Neuropsychological Tests

We conducted a randomized, controlled design to determine the effect of cognitive training on brain function and cognitive function (Cheng et al., 2012). The neuroimaging data and cognitive measurement were collected at baseline and at 1 year after training.

The multi-domain training group received 24 sessions (each session was 60 min) of cognitive training at a frequency of twice a week over a 12-week period, and the training procedure took place in a classroom in Tongji Hospital. The multi-domain cognitive training targeted memory, reasoning, problem-solving strategies, visual-spatial map reading skill developments, handcraft making, and health and physical exercise. A lecture about a common disease in aging people was presented during the first 15 min of each session. Then, the trainer taught the participants about a special cognitive strategy or technique via slide lecture during the second 30 min. The newly practiced skills were consolidated by dealing with some real-life problems during the last 15 min.

The wait-list control group served as a match for the social contact associated with cognitive training. The multi-domain training group and the control group attended a lecture about healthy living every 2 months (the training details are found in our previous study; Cheng et al., 2012).

To evaluate the effects of intervention on cognitive function, all measurements were administered at baseline and at 1 year after intervention, including the Repeatable Battery for the Assessment of Neuropsychological Status (RBANS, Form A), which has good validity and reliability in a Chinese community-living elderly sample (Lim et al., 2010; Cheng et al., 2011), the trail making test (TMT; Ashendorf et al., 2008), the visual reasoning test (Xiao et al., 2002) and the Color Word Stroop test (CWST; van Boxtel et al., 2001).

### Data Acquisition

All participants were scanned using a Siemens 3T MRI Scanner (Erlangen, Germany) at baseline and at 1 year after training ending at East China Normal University, Shanghai, China. To minimize head motion, foam pads were used to fix the subjects’ heads. High-resolution T1-weighted images were acquired using a magnetization-prepared rapid gradient-echo sequence, generating 160 slices (repetition time (TR) = 1900 ms, echo time (TE) = 3.43 ms, flip angle (FA) = 90 degrees, matrix size = 256 × 256, field of view (FOV) = 240 × 240 mm^2^, slice thickness = 1 mm; no gap). Resting state functional images were acquired using a single-shot, gradient-recalled echo planar imaging sequence (TR = 2000 ms, TE = 25 ms and FA = 90 degrees, FOV = 240 × 240 mm^2^, matrix = 64 × 64, slice thickness = 5 mm (no gap), 32 slices per volume). All subjects underwent a 310 s scanning to yield a total of 155 volumes. The subjects were instructed to rest with their eyes closed, not to think of anything in particular, and not to fall asleep.

### Data Pre-process Analysis

Pre-processing of fMRI data were conducted using the SPM8 Software Package [statistical parametric mapping^1^]. The slice time correction, 3D motion detection and correction, spatial normalization and resample (3 mm × 3 mm × 3 mm), and spatial smoothing using an isotropic Gaussian kernel (6 mm full width at half maximum) were applied. The processing is identical to that used in prior studies (Chen et al., 2015; Jiang et al., 2016). Only the subjects with head motion less than 1.5 mm and 1.5° during fMRI acquisition were included in the following preprocessing. In addition, the translation and rotation of the subjects were assessed by frame wise displacement (FD),

$$FD_{i}=\left|\Delta d_{ix}\right|+\left|\Delta d_{iy}\right|+\left|\Delta d_{iz}\right|+\left|\Delta \alpha_{i}\right|+\left|\Delta \beta_{i}\right|+\left|\Delta \gamma_{i}\right|,$$

where *i* is the *i*-th time point, and $\Delta d_{ix}=d_{(i-1)x}-d_{ix}$ (similarly for the other head motion/rotation parameters; Power et al., 2012). No significant differences were found between groups in FD. Additional preprocessing in preparation included voxelwise nuisance correction by regressing out six motion signals.

### ICA Decomposition

Similar to the approaches in our previous studies (Luo et al., 2012c; Li et al., 2015b), we first conducted spatial group ICA in which two times of data of all subjects were included in one group, using the GIFT Software, version 2.0a^2^. The time courses were temporally concatenated across subjects and reduced by means of principal component analysis in temporal domain, followed by an IC estimation using the FastICA algorithm. This algorithm was repeated 20 times in ICASSO^3^, and the resulting components were clustered to estimate the reliability of the decomposition (Himberg et al., 2004). Dimension estimation on all subjects was performed using the minimum description length (MDL) criterion to determine the number of independent components (ICs). The ICs were back-constructed for each scans and each subject using the dual-regression approach (Zhang et al., 2010). Finally, we interrogated all components to identify RSNs. Ten RSNs were chosen based on the average power spectra and spatial map of the components such as in the previous studies (Luo et al., 2012c; Li et al., 2015b). Here, the power spectra of time course of ICs was analyzed, and the power spectra of the selected IC should show the frequency content was mainly concentrated below 0.1 Hz (Beckmann et al., 2005; Luo et al., 2012b).

### Spatial Normalization to Symmetric Templates

To acquire the hemispheric symmetries’ RSNs, the warping step was performed from the resulted RSNs to the symmetries’ templates. First, the MNI template was warped to a symmetrized version of the MNI template because all subjects’ functional images were normalized into the MNI template. Then, the MNI template was normalized to the symmetrized template. Finally, the warping parameters were applied to all components for the symmetrizing RSNs in all subjects.

### Calculation of Voxelwise Homotopic Maps

The component values were initially normalized across voxels using *z*-score. The differences (*B*_v_) between component values of both homotopic voxels on both sides of cerebral cortex were calculated using the following formula 1. As with our previous study (Agcaoglu et al., 2015), the difference was shown in a map with a positive difference (*R* > *L*) on the right side of the brain and negative difference (*L* > *R*) on the left side of the brain.

(1) $$B_{v}=\{\text{​​​}\begin{array}{c} \left(R_{v}-L_{v}\right)ifR_{v}>L_{v}\\ \left(L_{v}-R_{v}\right)ifL_{v}>R_{v}\\ 0\;otherwise \end{array}$$

where *R* and *L* represents the right hemisphere and left hemisphere for each pairs of homotopic voxels *v*.

Hence, the voxelwise homotopic map was produced for each subject each RSN.

### Laterality Cofactor

The laterality cofactor (LCF) was defined based on a voxelwise homotopic map, as in the previous study (Agcaoglu et al., 2015). The amount of laterality for a given RSN was calculated in the global laterality metric. The LCF was acquired by taking the differences between the sum of all intensities of laterality on the right and left hemispheres with respect to the sum of all intensities across the brain (Formula 2). The LCF was computed for the average models for each group. In addition, individual LCF was calculated to evaluate the alteration of LCFs related with training.

(2) $$\text{LCF}=\frac{S_{l}-S_{r}}{S_{l}+S_{r}}$$

Where *S_l_* and *S_r_* represents the sum of all intensities of laterality on the left and right hemispheres.

### Statistical Test

For each of the components, one sample *t*-test was performed in voxelwise homotopic maps in three groups. Here, the pretraining fMRI data from all the subjects were included in one group; the second data were divided into training and control groups on the basis of receiving the training or not. For the three *t*-value maps, we then applied a mask to retain those voxels whose *t*-values exceed one standard deviation of the *t*-value across voxels. The thresholded *t*-maps were used to compute the LCFs of group level (Global LCF) according to the same formula to calculate the LCF (Formula 2).

According to the formula 2, the LCF value range was between −1 and 1. The Fisher-*z*-translation of individual LCF was performed for the distribution normality for each RSN. The individual LCF of each RSN was also evaluated used the ANOVA for repeated measurement data with *post hoc*, pair-wise Scheffe test in SPSS v20 (IBM Corp., Somer, NY, USA).

## Results

### Demographic Information

At baseline, 23 subjects from the multi-domain training group and 17 subjects from the control group underwent cognitive measurements and fMRI scanning. Eighteen of the multi-domain training group and 14 of the control group finished the cognitive measurements and fMRI scanning at 1 year after the intervention. A total of eight participants withdrew, one due to death, two due to intestinal cancer, one due to her husband’s death, one due to operation and three due to rejecting the scanning. No significant differences with regard to age, gender, education and MMSE score were found between the multi-domain training group and the control group (Table 1).

**Table 1:** **Demographic information of the subjects**.

		Multi-domain training group	Control group	*p*
Age (year)	Baseline	70.61 ± 3.29	68.59 ± 3.24	0.838
	One-year Posttest	72.39 ± 3.43	70.85 ± 4.05	0.782
Gender (male)	Baseline	23 (16)	17 (9)	0.283
	One-year Posttest	18 (13)	14 (9)	0.631
Education (year)	Baseline	10.91 ± 3.65	10.64 ± 3.06	0.452
MMSE	Baseline	27.57 ± 2.57	28.17 ± 1.94	0.505
	One-year Posttest	27.72 ± 2.16	27.85 ± 2.31	0.900

### Identification of RSN and Voxelwise Homotopic Maps

**Figure 1:**
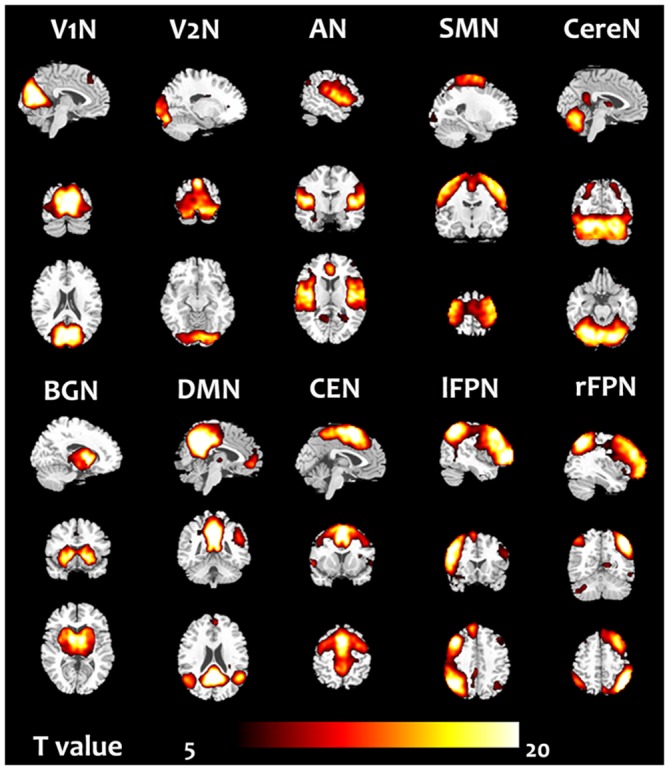
**Ten resting state networks (RSN) were chosen.** The one sample *t*-test in the group included all subjects in the pre-training scans was performed.

There were 45 components resulting from the group ICA. Ten components were selected as nonartifactual, relevant networks by visual inspection in accordance with published results (Luo et al., 2012c; Li et al., 2015b). The spatial maps of these RSNs were highly stable (reliability index >0.83) as determined by ICASSO. The 10 RSNs were shown in Figure 1 and labeled as follows:

V1N:  primary visual network. The visual cortex is often apparent in two separate components. This network showed spatial patterns consisting of primary visual area (Damoiseaux et al., 2006; Luo et al., 2012c).
V2N:  the second visual network illustrated spatial patterns consisting of more lateral visual arear in the occipital lobe which were previously known to be involved in visual processing (Damoiseaux et al., 2006; Luo et al., 2012c).
AN:  auditory network (AN) primarily encompassed bilateral middle, superior temporal gyrus, temporal pole and insular, corresponded to the auditory system (Damoiseaux et al., 2006; Luo et al., 2012c).
SMN:  sensorimotor network (SMN) was a network corresponding to sensory-motor function. This network includes pre- and postcentral gyrus, the primary sensorimotor cortices, and the supplementary motor area (Damoiseaux et al., 2006; Luo et al., 2012c).
CereN:  cerebellum network included bilateral cerebellum hemispheres.
DMN:  default mode network (DMN) mainly encompasses posterior cingulate cortex, medial prefrontal gyrus, bilateral superior frontal gyrus, and bilateral angular gyri (Raichle et al., 2001; Damoiseaux et al., 2006; Luo et al., 2012c).
lFPN:  left lateral FPN (lFPN) along with right lateral FPN showed the similar spatial patterns with DAN consisting of regions previously known to be involved in goal-directed top-down processing (Damoiseaux et al., 2006; Luo et al., 2012c). This network primarily involved precuneus, inferior parietal lobule, middle frontal gyrus, superior parietal lobule.
rFPN:  right lateral FPN (rFPN) including clusters lateralized to the right hemisphere putatively associated with DAN. lFPN and rFPN were the only maps to be strongly lateralized and were largely left–right mirrors of each other (Damoiseaux et al., 2006; Luo et al., 2012c).
CEN:  central executive network showed spatial patterns consisting of the superior and middle prefrontal cortices, anterior cingulate and paracingulate gyri, and ventrolateral prefrontal cortex (Beckmann et al., 2005).
BGN:  basal ganglia network, encompassed middle temporal gyrus, superior temporal gyrus, insular and temporal pole and corresponded to the auditory system (Luo et al., 2012b).

**Figure 2:**
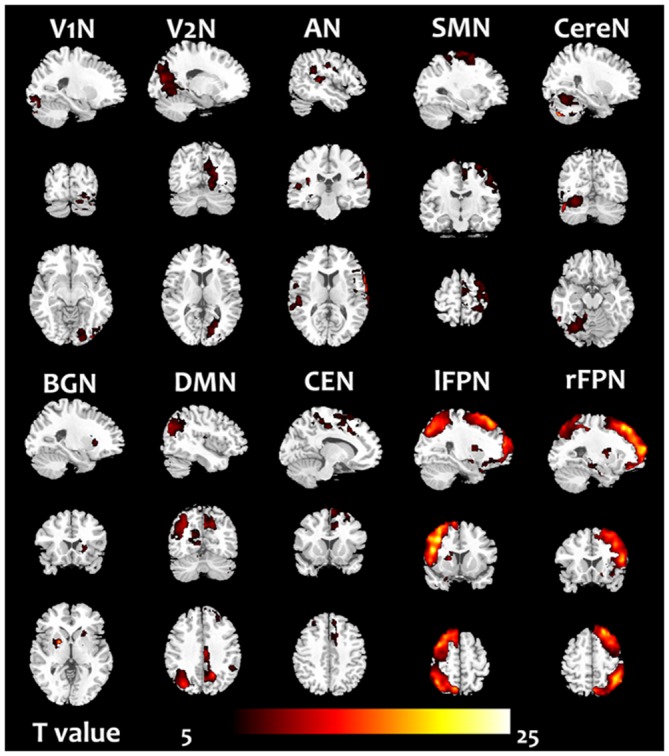
**The homotopic maps of the 10 RSN.** The group level maps resulted from one sample *t*-test in the groups including the pre-training scans (before cognitive training) of all subjects.

For 10 RSNs, their voxelwise homotopic maps were calculated for each group. Because the homotopic maps were similar to each other among groups, the group level maps resulted from the pre-training scans were illustrated in the Figure 2.

### Laterality Cofactors

**Figure 3:**
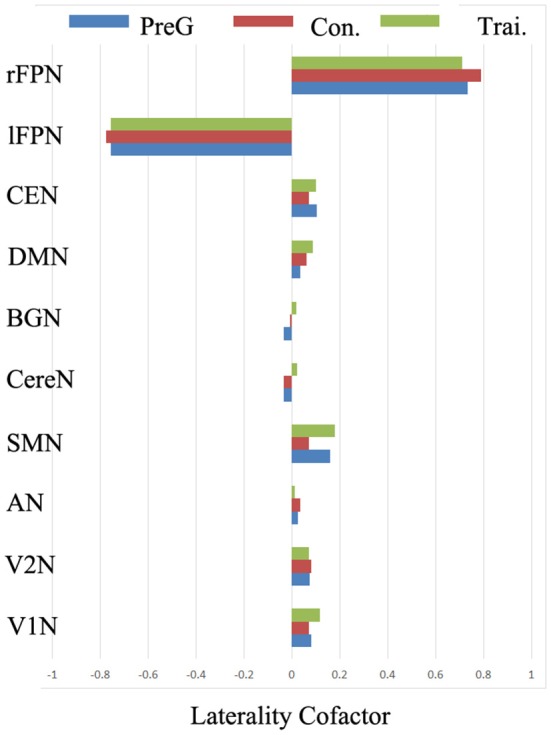
**The group level laterality cofactor (LCF) resulted from the three group *Bv* homotopic maps for each RSN.** Three groups included the pre-training group including all subjects, the post-training group and the control group of the second scans.

In the three groups, the global LCF of each RSN were illustrated in Figure 3. According to the previous criteria (Agcaoglu et al., 2015), the LCFs are called highly lateralized when it has absolute value above 0.75 or has lateralized with absolute value above 0.2; the LCFs in rFPN and lFPN were highly lateralized in three groups. The remaining eight RSNs did not have marked lateralization, in which the LCFs have absolute value less than 0.2.

**Figure 4:**
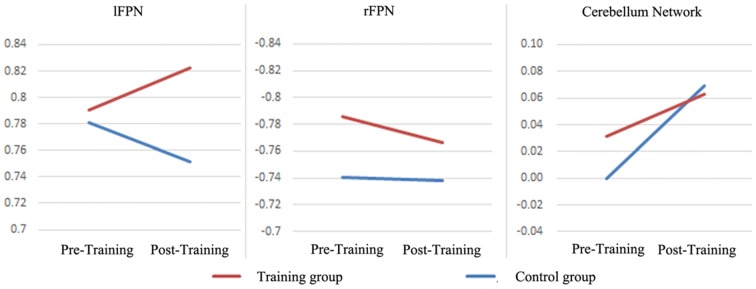
**The LCFs of individual *Bv* maps in the lFPN, rFPN and CereN.** The repeated measure ANOVA were performed. The significance of statistical test was demonstrated in the Table 2.

To investigate the influence of the multiple cognitive training on the laterality of RSN, the LCFs of each RSN and each subject were also evaluated (Table 2, Figure 4). After the Fisher *r*-to-*z* translation, a repeated measure ANOVA revealed that both groups improved their LCFs in CereN as indicated by a significant main effect of time (*F* = 6.903, *p* = 0.015). In addition, a significant training main effect on the LCF in rFPN (*F* = 5.897, *p* = 0.021) and lFPN (*F* = 7.641, *p* = 0.01), as well as interactions of LCF of lFPN (*F* = 8.908, *p* = 0.006), were observed. *Post hoc* analysis showed significantly increased LCF of lFPN in the training group compared with the control group at the second scan (*T* = 3.48, *p* = 0.001). Paired-sample *t*-tests revealed that the global LCF of CereN (*T* = 2.30, *p* = 0.03) was significantly increased after the training scan compared with the before-training scan in the control group but not in the training group. Similarly, compared with the before-training scan, the after-training scan showed the decreased LCF of lFPN (*T* = 2.21, *p* = 0.04) in the control group, whereas a marginal significant increase (*T* = 2.02, *p* = 0.06) was observed in the training group.

**Table 2:** **The results of the repeated measure ANOVA for the laterality cofactors of individual *Bv* maps in 10 RSNs**.

		V1N	V2N	AN	SMN	CereN	BGN	DMN	CEN	lFPN	rFPN
Time main effects	*F* value	3.498	0.279	0.111	3.888	6.903	1.044	0.677	0.901	0.006	1.183
	*P* value	0.071	0.601	0.741	0.058	0.015*	0.315	0.417	0.35	0.939	0.285
Training main effects	*F* value	0.192	0.264	0.048	0.699	0.499	3.808	1.088	0.026	7.641	5.897
	*P* value	0.664	0.611	0.828	0.41	0.485	0.06	0.305	0.872	0.01*	0.021*
Interaction effects	*F* value	0.229	0.012	0.821	1.858	0.945	0.27	1.057	0.102	8.908	0.649
	*P* value	0.636	0.915	0.372	0.183	0.339	0.607	0.312	0.752	0.006*	0.427

Note: *Represented the statistical significance p < 0.05.

## Discussion

The lateralization of human brain function is an obvious sign reflecting functional specialization. Neurodegeneration would alter the functional specialization in aging. In this study, we used the LCF (Agcaoglu et al., 2015) of intrinsic networks resulting from the resting fMRI to investigate the effects of multi-domain cognitive training on healthy older adults. We observed statistically significant training effects on the lateralization of two important RSNs related to high-level cognition: right- and left- FPNs. In particular, the lateralization of lFPN were retained well in the training group but decreased in the control group. The increased lateralization with aging was observed in the CereN, in which the lateralization was significantly increased in the control group, although the same change tendency was observed in the training group. These findings indicate that the lateralization of the high-level cognitive intrinsic networks is sensitive to multi-domain cognitive training. This study provides neuroimaging evidence to support the idea that cognitive training should have advantages in preventing the cognitive decline in healthy older adults.

Both human and animal studies indicates neural plasticity across the lifespan (Ball et al., 2002; Papp et al., 2009). A number of studies support the protective effects of late-life cognitive training on dementia (Ball et al., 2002; Wilson et al., 2002; Snowball et al., 2013). Our previous study has illustrated positive effects of multi-domain cognitive training interventions in healthy older adults. Here, the findings from neuroimaging were provided to uncover the potential brain changes to response the effects of interventions. The functional neuroimaging biomarkers can play an important role in detecting, assessing and diagnosing neurodegeneration (Horwitz and Rowe, 2011). In addition, there was altered intrinsic connectivity in the special targeted networks such as DMN and salience network to different neurodegenerative disorders (Seeley et al., 2009). Recently, the lateralization of RSNs was observed as an alteration accompanied by increased aging (Wilson et al., 2002). The hemispheric lateralization was also associated with enhanced cognitive ability (Gotts et al., 2013). Thus, the spatial pattern of intrinsic networks would be a candidate feature for the cognitive training intervention in older adults. Enhancement and maintenance of memory, visuospatial/construction and attention endured in healthy older adults with multi-domain cognitive interventions (Cheng et al., 2012). The changed lateralization of FPN and CereN which was observed in the cohort might be related to the brain mechanism of these behavior improvements.

The FPNs are associated with attention, cognitive performance and control processes. In the studies of resting state fMRI, the FPN is often identified using ICA. FPNs have marked asymmetry, which is involved in a multitude of cognitions; the right is more involved in monitoring processes whereas the left is putatively more involved in production processes (Cabeza et al., 2003). Combining lFPN and rFPN showed a similar spatial pattern, with a dorsal attention network consisting of regions previously known to be involved in goal-directed top-down processing (Damoiseaux et al., 2006; Luo et al., 2012c). Significant interactions of global LCF of lFPN were observed in this study. Global LCF of lFPN was significantly decreased in healthy controls whereas a marginal significant increase was observed in the training group. We presumed that the lateralization of lFPN would be an important predictor for maintenance of attention functioning and production processes. These findings implicated that the multi-domain cognitive training would contribute to the top-down attention function in the healthy older adults. Although the training main effects were also observed in the rFPN using repeated measure ANOVA, as indicated by the difference between the training group and control group, the training main effects might be stained by the initial distinction between groups (Figure 4). Thus the influence of training on the rFPN would not be evaluated directly in this study. In general, the FPN would be a target to multi-domain cognitive training.

Motor-related function decline is another physiological sign in old adults compared with younger adults. Accumulating evidence demonstrates decreased functional connectivity in the motor-related networks in aging, including the CereN and sensory-motor network (Tomasi and Volkow, 2012). Consistent with the previous studies, we found that the CereN is symmetrical at baseline (first scanning). However, improved lateralization of the CereN was observed in both group as indicated by a significant main effect of time in repeated measure ANOVA. The laterality significantly increased between two times of scans with interval of 1 year in healthy control groups whereas it did not in the training group. This finding reflected that the symmetry of the CereN was maintained in healthy older adults with multi-domain cognitive training. Recently, the functional connectivity studies indicate that the cerebellum participates in functional networks with motor control and cognitive processes (Habas et al., 2009; Krienen and Buckner, 2009). Thus, our findings might implicate that the multi-domain cognitive intervention contributed to the improvement of motor control in older adults. Actually, SMN would be directly related with motor performance (Zhang et al., 2015). Seidler et al. (2015) found greater network interactivity of SMN in older adults and suggested the protection against age declines in motor performance. However, no difference of the lateralization was found in the SMN in this study. It did not mean that the negative effects of cognitive training on the motor performance and motor prediction which are related with SMN are responsible. Future studies should focus on the various aspects of motor function associated with the cognitive intervention.

There are several limitations which should be mentioned here. First, the longitudinal design with 1-year duration was adopted in this study. The subjects were randomly grouped, and the behavior scores and genders were matched between groups. However, some connectivity features from the resting state fMRI might not match because their features could be calculated according to different methods. Thus, some features whose difference illustrated by repeated measure ANOVA might be stained by the mismatch in baseline (the first time) measures. Second, the relative small sample size would be another shortage of our study. In the future, more subjects should be recruited into the longitudinal study. Finally, the Group ICA was used to investigate the symmetry of the spatial pattern of RSNs. However, the number of ICs might be an underlying confounding factor. Till now, there has been no validated way to identify the number of ICs in ICA analysis. In this work, the MDL criteria implanted in GIFT software was used to determine the number of ICs.

In conclusion, lateralization of function is an important organization of the human brain. The distribution of intrinsic networks in the resting brain and their association with the multi-domain cognitive training is analyzed in this study. The lateralization of bilateral FPN and CereN was related to both aging and the multi-domain cognitive training in healthy older adults. These findings provide a neuroimaging evidence to support the positive effects of the cognitive training on enhancement and maintenance cognitive and motor function in healthy older adults.

## Author Contributions

Conceived and designed the work: CLi, CLuo and DY; Acquired the data: XC, TL, YC and LJ; Analyzed the data: WC, XZ, CLuo, YG, XC and YC; Wrote the article: CLuo, CLi and DY. All authors revised the work for important intellectual content. All of the authors have read and approved the manuscript.

## Conflict of Interest Statement

The authors declare that the research was conducted in the absence of any commercial or financial relationships that could be construed as a potential conflict of interest.

## Footnotes

^1^http://www.fil.ion.ucl.ac.uk/spm

^2^http://icatb.sourceforge.net/

^3^http://research.ics.tkk.fi/ica/icasso
